# Supplementary material for: Dowker complex based machine learning (DCML) models for protein-ligand binding affinity prediction
Source: PLoS Comput Biol. 2022 Apr 6;18(4):e1009943. doi: 10.1371/journal.pcbi.1009943 (PMC8985993; doi:10.1371/journal.pcbi.1009943)
Supplement: Attachment — Submitted filename: Reply_Letter.pdf [file pcbi.1009943.s001.pdf]

Dr Kelin Xia
School of Physical and Mathematical Sciences
School of Biological Sciences
Nanyang Technological University
Singapore 637371
Email: xiakelin@ntu.edu.sg
Phone: +65 6513 7464

December 5, 2021

Prof. Arne Elofsson
On behalf of PLOS Computational Biology

Submission ID: PCOMPBIOL-D-21-01678

Dear Prof. Arne Elofsson,

Thank you very much for your email on October 12, 2021 and referees' reports concerning our paper "Dowker complex based machine learning (DCML) models for protein-ligand binding affinity prediction" (submission ID: PCOMPBIOL-D-21-01678). We have carefully addressed all the concerns from reviewers and revised our paper accordingly. We sincerely believe our revised version is suitable for PLOS Computational Biology.

Sincerely yours,

Kelin Xia
Assistant Professor

Answers to Reviewer 1' Comments

Comments: *Here the authors proposed a Dowker complex based molecular interaction representations, which used a bipartite graph to model the interactions between a protein and a ligand. Then a DC-based persistent spectral model was constructed and the persistent Riemann Zeta functions were calculated as molecular descriptors. Finally, a DC-based gradient boosting tree model was trained to predict protein-ligand binding affinity. This is a novel method to represent protein-ligand interaction as bipartite graph and calculate the descriptors from the knowledge of topology and graph theory. When it was applied to protein-ligand affinity prediction, however, I have some concerns about the representations and models:*

Answer: We thank the reviewer for his/her supportive comments.

Comments: *1. In order to calculate the descriptors, the binding core region was defined using a cutoff distance of 10Å. I wonder how you defined the cutoff. Actually, I have seen some different definitions about the binding core region, ranging from 5Åto 12Å. Does the cutoff distance influence the results a lot?*

Answer: We thank the reviewer for pointing out this. We have not tested the different cutoff distances and their effects for our model. We believe if a very small cutoff distance such as 5 Å is used, there will be some influence for the model.

In this paper, we use exactly the same cutoff distance as in the PDBbind databases, so that a fair comparison with all other models can be achieved. In fact, PDBbind datasets have already provided separated files for the binding core regions (to help with a fair comparison between scoring functions).

Comments: *2. According to the manuscript, the size of feature vectors depend on the filtration values and the number of Riemann Zeta functions. Did they have physical or mathematical significance? Or were they selected by hyper-parameter optimization?*

Answer: We thank the reviewer for pointing out this. The filtration process is used for the generation of a multiscale representation. In general, a small filtration value covers only local interactions, while a large filtration value characterizes long-range interactions. The Riemann Zeta functions play a pivotal role in analytic number theory, and has applications in physics, prob-

ability theory, and applied statistics. Roughly speaking, the higher-order terms of Riemann Zeta functions characterize complicated nonlinear interactions. The Riemann Zeta functions are used for molecular featurization in our model.

In our model, the filtration parameter is chosen exactly as the cutoff distance. We have tested different filtration values, and our model is comparably robust, even though a very large filtration value will result in a much larger computational cost. Further, the order for Riemann Zeta functions is chosen as integers from -5 to 4. It has been found that further enlargement of the function order will not greatly improve the results. As a balance of computational cost and model accuracy, we have chosen the above values.

We totally agree that if these parameters are regarded as hyper-parameters, a hyper-parameter optimization will further increase the accuracy of our model. However, the main focus of the current paper is the introduction of these new mathematical models. The parameter optimization will be fully explored in later papers.

Comments: *3. In page 8/17, line 226: "Note that the accuracy of our DC-based models can be further improved if convolutional neural network models, such as the one used in TopBP models" Have you already tried the convolutional neural network models or you just imagined that?*

Answer: We have already tested CNN-based models and found that they can improve the final prediction results. The findings are consistent with the results in TopBP model, which also uses CNN. However, a key issue is how to transfer features into suitable 1D or 2D data, that is suitable for CNN models. We have proposed molecular persistent spectral image (Mol-PSI) representation by transforming the spectral information into 1D and 2D image data. The combination of these data with CNN models can further improve the results. The paper is currently under review.

Comments: *4. The Table 1 listed the detailed information of the three PDBBind databases. I noticed that the Training set includes all the remained data when removing Test set from Refined set. Is there a validation set when you train your model? And how the hyper-parameters listed in Table 2 were selected?*

Answer: We thank the reviewer for pointing out this. Indeed, the Training

set includes all the remained data when removing Test set from Refined set. No validation set is used to train our model. In fact, the hyper-parameters are NOT optimized and are simply taken from our previous model,

Meng, Zhenyu, and Kelin Xia. "Persistent spectral-based machine learning (PerSpect ML) for protein-ligand binding affinity prediction." Science Advances 7.19 (2021): eabc5329

Indeed, a further hyper-parameter optimization can improve the accuracy of the current model.

As stated above, the main focus of this paper is to introduce new mathematical models and tools for protein-ligand binding affinity prediction. We believe that topological representations and featurization are of key importance for the problem. Innovative mathematical models can bring new insights into biomolecular analysis.

Comments: *5. There are many different type of protein-ligand affinity prediction models, which can also be called scoring functions. The scoring power is not the only problem we concerned, there are test sets for testing the docking power and screening power in CASF-2016 (or other version). We are very interested in the docking power and screening power of the model. We suggested that you provide the related results.*

Answer: Following the reviewer's suggestions, we have tested the docking power and screening power of our model on benchmark CASF-2013. We have followed the common procedures to generate the docking poses. The docking power is tested based on the 195 ligand cases, and screening power is tested based on 65 different proteins. Our model can achieve the state-of-the-art results for both docking power and screening power tests. We have added two more sections in the revised version. More details for the comparison of our model with other models can be found in Figure 4.

Comments: *6. In page 8/17, line 231: "We do not compare with these models because the training and testing sets of these models are different from the standard ones in PDBbind datasets". Considering that all the PDBBind datasets are public, it is not difficult to make a comparison. I think more evidence should be given to prove the advantage of DC-based molecular interaction representations.*

Answer: We thank the reviewer for pointing out this. We have revised it

accordingly. In fact, we have already done the comparison and listed all the results for these models in Table 5. We do not list these results in Figure 3 to avoid an unfair comparison. Note that all models in Figure 3 use exactly the same training and testing datasets, so that a fair comparison is possible. The models listed in Table 5 use a much larger training set to boost the performance of their model.

Answers to Reviewer 2' Comments

Comments: *This work proposes novel molecular descriptors for protein-ligand binding affinity predictions. These descriptors are constructed from Dowker complex and spectral graph information. The authors have validated the robustness and the efficiency of the proposed features against series of PDBbind benchmarks. Overall this manuscript is well-written and easy to follow. Besides these positive sides, there are some downsides I would like to bring up here*

Answer: We thank the reviewer for his/her supportive comments.

Comments: *1) Proposed models use charges, distances, DC-based features, etc. General readers will appreciate it if authors carefully investigate the performances of the separated features. There might be some redundant features.*

Answer: We thank the reviewer for pointing out this. The performance of the separated features is listed in Table 3, in which we have three models with different features, i.e., Dist-based features, Charge-based features, and combined features. In general, distance-based features and charge-based features tend to characterize different aspects of molecular properties. Even though there should still be some "overlapping" between the two types of features, the random forest learning models are comparable robust and can take care of some "redundant" features.

Comments: *2) I do not know how atom charges were obtained. Please provide such a discussion in the revised version.*

Answer: We thank the reviewer for pointing out this. We use the software "PDB2PQR",

Dolinsky TJ, Czodrowski P, Li H, Nielsen JE, Jensen JH, Klebe G, et al. "PDB2PQR: Expanding and upgrading automated preparation of biomolecular structures for molecular simulations". Nucleic Acids Res. 2007;35:W522525.

We have added some more discussions and cited the reference.

Comments: *3)Lines 226-228, authors claim that CNN can further improve their current model. Are there any hard proofs? If yes please provide them otherwise I suggest removing these sentences.*

Answer: We thank reviewer for pointing out this. As stated above, we have already tested CNN-based models and found that they can improve the final prediction results. The findings are consistent with the results in TopBP model, which also uses CNN. However, a key issue is how to transfer features into suitable 1D or 2D data, that is suitable for CNN models. We have proposed molecular persistent spectral image (Mol-PSI) representation by transforming the spectral information into 1D and 2D image data. The combination of these data with CNN models can further improve the results. The paper is currently under review.

Comments*: 3) Please include TopBP in figure 3 since it is discussed in Table 4*

Answer: We thank the reviewer for pointing out this. We have redraw the Figure 3 as in the manuscript.

Comments*: 4) There are missing data files/features files from the authors provided Github link. Please update them.*

Answer: We have added accordingly.
