# Supplementary material for: Dowker complex based machine learning (DCML) models for protein-ligand binding affinity prediction
Source: PLoS Comput Biol. 2022 Apr 6;18(4):e1009943. doi: 10.1371/journal.pcbi.1009943 (PMC8985993; doi:10.1371/journal.pcbi.1009943)
Supplement: Attachment — Submitted filename: Reply_Letter_v2.pdf [file pcbi.1009943.s003.pdf]

Dr Kelin Xia
School of Physical and Mathematical Sciences
School of Biological Sciences
Nanyang Technological University
Singapore 637371
Email: xiakelin@ntu.edu.sg
Phone: +65 6513 7464

February 13, 2022

Profs. Joanna Slusky and Arne Elofsson
On behalf of PLOS Computational Biology

Submission ID: PCOMPBIOL-D-21-01678R2

Dear Profs. Joanna Slusky and Arne Elofsson,

Thank you very much for your email on January 31, 2022 and referees' reports concerning our paper "Dowker complex based machine learning (DCML) models for protein-ligand binding affinity prediction" (submission ID: PCOMPBIOL-D-21-01678R2). We have carefully addressed all the concerns from reviewers and revised our paper accordingly. We sincerely believe our revised version is suitable for PLOS Computational Biology.

Sincerely yours,

Kelin Xia
Assistant Professor

Answers to Reviewer 1' Comments

Comments: *The authors have responded adequately to most of my comments, but there are some problems in the response to the question about docking power and screening power. The scoring, docking and screening powers should be evaluated using the same scoring function model, but the authors retrained their model for each ligand in the docking power test and each protein in the screening power test. The performance of these target-specific scoring models cannot be compared to the performance of those general scoring functions listed in Figure 4, except the AGL-Score. In other words, the model which is used to evaluate the docking power and screening power should be the same model that is used to evaluate scoring power.*

Answer: We thank the reviewer for pointing out this. Indeed, it will be great to have a general scoring function that works for all the scoring, docking and screening tests. Similar to AGL-score model, our DCML is a target-specific scoring model. For different tasks, different training datasets are considered in our model. Following the reviewer's suggestion, we do not directly compare our models with the existing general-scoring-function-based models, and we have removed Figure 4 to avoid confusion.

Currently, it is challenging to design a general scoring function with high accuracy. Target-specific scoring models have been developed by using different training sets for different tasks, and have demonstrated a great potential. In particular, decoy data has been incorporated into training set for the improvement of docking power. For example, in $\Delta_{Vina}RF20$, the training set consists of 3336 experimental structures and 3322 computer-generated decoy structures. In DeepBSP, the model is trained on a total of 177300 poses including both experimental and decoy data. In NNScore, 287 poorly docked protein-ligand complexes are added into the training set. In *smina*, computer-generated ligand poses are used in the training set.

Below we has listed several target-specific scoring function models using different datasets. The key sentences are marked in green color.

1. Bao J, He X, Zhang J Z H. DeepBSP:a machine learning method for accurate prediction of protein-ligand docking structures[J]. Journal of Chemical Information and Modeling, 2021, 61(5): 2231-2240.

DeepBSP—a Machine Learning Method for Accurate Prediction of Protein–Ligand Docking Structures

Jingxiao Bao, Xiao He,* and John Z. H. Zhang*

Cite This: *J. Chem. Inf. Model.* 2021, 61, 2231–2240 Read Online

ACCESS | Metrics & More | Article Recommendations | Supporting Information

ABSTRACT: In recent years, machine-learning-based scoring functions have significantly improved the scoring power. However, many of these methods do not perform well in distinguishing the native structure from docked decoy poses due to the lack of decoy structural information in their training data. Here, we developed a machine-learning model, named DeepBSP, that can directly predict the root mean square deviation (rmsd) of a ligand docking pose with reference to its native binding pose. Unlike the binding affinity, the rmsd between the docking poses with reference to their native structures can be straightforwardly determined. By training on a generated data set with 11,925 native complexes and more than 165,000 docked poses, our model shows excellent docking power on our test set and also on the CASF-2016 docking decoy set compared to other major scoring functions. Thus, by combining molecular dockings that generate many poses with the application of DeepBSP, one can more accurately predict the best binding pose that is closest to the native complex structure. This DeepBSP model shall be very useful in picking out poses close to their natives from many poses generated from a dock application.

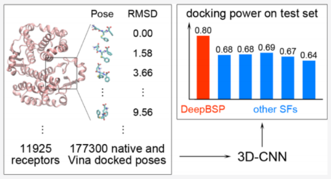

2. Wang C, Zhang Y. Improving scoringdockingscreening powers of proteinligand scoring functions using random forest[J]. Journal of computational chemistry, 2017, 38(3): 169-177.

From the above discussion, we can see that the inferior performance of RFbScores in docking tests may reflect two problems: (1) Random forest is restricted to do interpolation; (2) Use of experimental protein–ligand binding data with known crystal structures as the training set alone limits the applicability of RFbScores. In this work, to improve scoring-docking-screening powers of scoring functions simultaneously, we employ a two-pronged strategy:

One is to expand the training set. Besides enlarging the experimental dataset to include crystal structures with weak binding affinities, we have added a similar amount of computationally generated structures (decoy data) into the training set. The idea of including decoy data in the training set has been previously employed in the development of several other scoring functions.[34,37]

[illegible]

3. Ragoza M, Hochuli J, Idrobo E, et al. Proteinligand scoring with convolutional neural networks[J]. Journal of chemical information and modeling, 2017, 57(4): 942-957.

Training Sets. We utilize two training sets focused on two different goals: pose prediction and virtual screening. In all cases we generate ligand poses for actives and decoys using docking with smina[1] and the AutoDock Vina scoring function.[7] We note that methods using the AutoDock Vina scoring function performed well in blind evaluations of docking performance.[46,47] We use docked poses, even for active compounds with a known crystal structure, because (1) these are the types of poses the model will ultimately have to score and (2) to avoid the model simply learning to distinguish between docked poses and crystal structures (which were likely optimized with different force fields).

4. Li L, Wang B, Meroueh S O. Support vector regression scoring of receptorligand complexes for rank-ordering and virtual screening of chemical libraries[J]. Journal of chemical information and modeling, 2011, 51(9): 2132-2138.

There are a number of reasons as to why SVR methods or X-score did not show high enrichment levels with the DUD data sets. One possibility is that the training of SVR was performed on high-quality crystal structures of receptor−ligand complexes, while scoring in virtual screening is performed on docked structures that may or may not be the correct binding mode. To improve the performance of SVR-scoring methods in virtual screening, a similar strategy that was used to derive SVM-SP was followed. The success of SVM-SP was attributed to the fact that the scoring function was tailored to its target by including features obtained from complexes of decoy molecules docked to the target. Hence, the scoring function must be derived individually for each target. A similar strategy was used for SVR-KB. As shown in Table 5, the performance of the scoring function gradually improved with increasing number of decoys used in the training, eventually converging to <ROC-AUCs> = 0.71. We call this new scoring function SVR-KBD to reflect the inclusion of decoys in the training. While its performance did not exceed that of SVM-SP in virtual screening, SVR-KBD still showed higher enrichment than most scoring functions that were considered in this study.

5. Durrant J D, McCammon J A. NNScore: a neural-network-based scor-

ing function for the characterization of protein-ligand complexes[J]. Journal of chemical information and modeling, 2010, 50(10): 1865-1871.

Poorly Docked Database Preparation. In addition to the database of crystallographic and well-docked poses described above, we also generated a separate database of poorly docked protein−ligand complexes. Some of the ligands of the training/testing database described above were docked back into their corresponding receptors using AutoDock Vina.[23] Rather than identifying the ligand pose with the best predicted binding energy, the pose with the worst predicted binding energy was considered. In 287 cases, this worst predicted binding energy was greater than −4 kcal/mol; these 287 protein−ligand complexes were included in the poorly docked database.

6. Pham T A, Jain A N. Parameter estimation for scoring protein-ligand interactions using negative training data[J]. Journal of medicinal chemistry, 2006, 49(20): 5856-5868.

Parameter Estimation for Scoring Protein−Ligand Interactions Using Negative Training Data

Tuan A. Pham and Ajay N. Jain*

Cancer Research Institute, Department of Biopharmaceutical Sciences, and Department of Laboratory Medicine, University of California, San Francisco, 2340 Sutter Street, San Francisco, California 94143-0128

Received January 14, 2005

Surflex-Dock employs an empirically derived scoring function to rank putative protein−ligand interactions by flexible docking of small molecules to proteins of known structure. The scoring function employed by Surflex was developed purely on the basis of *positive* data, comprising noncovalent protein−ligand complexes with known binding affinities. Consequently, scoring function terms for improper interactions received little weight in parameter estimation, and an ad hoc scheme for avoiding protein−ligand interpenetration was adopted. We present a generalized method for incorporating synthetically generated *negative* training data, which allows for rigorous estimation of all scoring function parameters. Geometric docking accuracy remained excellent under the new parametrization. In addition, a test of screening utility covering a diverse set of 29 proteins and corresponding ligand sets showed improved performance. Maximal enrichment of true ligands over nonligands exceeded 20-fold in over 80% of cases, with enrichment of greater than 100-fold in over 50% of cases.

In our revised version, we have added some more discussions as follows.

Note that in our DC-GBT model, we consider target-specific scoring models and train them separatively for scoring, docking and screening power tests. Previously, general scoring function models were developed. That is for all the tasks, the same general scoring function is used. Recently, data-driven

learning models make use of the different types of training datasets to improve the performance of scoring functions. In particular, the incorporation of decoy data in the training set can significantly improve the docking and screening power [67-72]. In our DC-GBT models, different training sets are used, which results in different state-of-the-art scoring models in scoring, docking and screening power tests.

Answers to Reviewer 2' Comments

***Comments**: The authors have addressed all of my concerns.*

Answer: We thank the reviewer for his/her supportive comments.
